# Influence of the β^−^ Radiation/Cold Atmospheric-Pressure Plasma Surface Modification on the Adhesive Bonding of Polyolefins

**DOI:** 10.3390/ma14010076

**Published:** 2020-12-25

**Authors:** Martin Bednarik, Ales Mizera, Miroslav Manas, Milan Navratil, Jakub Huba, Eva Achbergerova, Pavel Stoklasek

**Affiliations:** 1 Faculty of Technology, Tomas Bata University in Zlin, Vavreckova 275, 760 01 Zlin, Czech Republic; jhuba@utb.cz; 2 Faculty of Applied Informatics, Tomas Bata University in Zlin, CEBIA-Tech, Nad Stranemi 4511, 760 05 Zlin, Czech Republic; mizera@utb.cz (A.M.); manas@utb.cz (M.M.); navratil@utb.cz (M.N.); achbergerova@utb.cz (E.A.); pstoklasek@utb.cz (P.S.)

**Keywords:** adhesion, radiation cross-linking, wetting contact angle, free surface energy, bonded joints, polypropylene, β^−^ radiation, plasma treatment

## Abstract

The goal of this research was to examine the effect of two surface modification methods, i.e., radiation cross-linking and plasma treatment, on the adhesive properties and the final quality of adhesive bonds of polypropylene (PP), which was chosen as the representative of the polyolefin group. Polymer cross-linking was induced by beta (accelerated electrons—β^−^) radiation in the following dosages: 33, 66, and 99 kGy. In order to determine the usability of β^−^ radiation for these applications (improving the adhesive properties and adhesiveness of surface layers), the obtained results were compared with values measured on surfaces treated by cold atmospheric-pressure plasma with outputs 2.4, 4, and 8 W. The effects of both methods were compared by several parameters, namely wetting contact angles, free surface energy, and overall strength of adhesive bonds. Furthermore, Fourier transform infrared (FTIR) spectroscopy and scanning electron microscopy (SEM) were conducted. According to our findings the following conclusion was reached; both tested surface modification methods significantly altered the properties of the specimen’s surface layer, which led to improved wetting, free surface energy, and bond adhesion. Following the β^−^ radiation, the free surface energy of PP rose by 80%, while the strength of the bond grew in some cases by 290% in comparison with the non-treated surface. These results show that when compared with cold plasma treatment the beta radiation appears to be an effective tool capable of improving the adhesive properties and adhesiveness of PP surface layers.

## 1. Introduction

In recent years, the use of polymer materials within the engineering fields requiring high technological standards, e.g., aviation and automotive industries, vastly expanded [1,2]. The reason for this occurrence could be the numerous advantages, for example low cost and weight, easy processability, and the possibility of recycling, which come with the application of polymer materials such as polyolefins [2,3,4,5,6].

Specific applications usually require one solid part, which can consist of individual pieces. One of the technologies that can be used to attach one polyolefin to another is adhesion bonding. This technology enables a creation of a bond, which provides several specific properties that are unobtainable with any other method. In comparison with mechanical bonding, such as welding and riveting, the usage of adhesive does not lead to the creation of stress and surface degradation within the unit [3]. Adhesive bonding, especially when used with polyolefins, also provides the following benefits [5,6,7,8,9,10,11]:

- Less stress on the interface of the bond, as the load is evenly distributed within a greater area, which leads to better integrity of the conjoined pieces.
- Vibration dampening, which increases rigidity and resistance to buckling.
- Significantly lower weight and superior adaptability to irregular surfaces in comparison to mechanical bonding.
- Ability to create water and gas tight bond, which disrupt neither the profile nor the aesthetics of the bonded part.

However, besides the aforementioned benefits, this technology of bonding also comes with several drawbacks and limitations, which have to be taken into account. The main limitation is the necessity to modify the surface layer before the bonding, so the wetting, energy, and adhesion of the surface are improved. The surface of polyolefins is generally considered to be quite difficult to modify. Nevertheless, a suitable technology enables the creation of controlled interface, whereby the required properties, such as compatibility and adhesion, can be obtained [9,12].

Surface treatment of the polyolefins, as well as the improvement of their adhesion properties, can be performed by numerous methods, e.g., corona discharge, flame/plasma treatment, or chemical etching [8,9,11]. Encinas et al. [13] have shown the positive effect of atmospheric-pressure plasma on the adhesion properties of high-density polyethylene (HDPE), low-density polyethylene (LDPE), and polypropylene (PP). This surface treatment lowered the contact angles of wetting, improved the wetting of the surface and increased the free surface energy and its polar component, which led to better adhesive properties. Furthermore, the improved properties remained stable in the tested time range (more than eight months). Likewise, the positive influence of the plasma upon the adhesive properties was proven in several other publications [1,14,15,16,17]. Kim et al. [18] demonstrated that the plasma has a positive effect not only on the adhesive properties, but also on the load bearing capabilities of the bonds. The results of the study proved the increasing shear strength of the adhesive bond when exposed to plasma with higher output and for longer duration. The main limitation of plasma treatment method is the aging phenomenon or hydrophobic recovery. These factors result from several physical and chemical processes, such as post-plasma oxidation (remaining radicals on the surface react with atmospheric air), post-plasma rearrangement or surface adaptation, and migration and diffusion of low molecular weight additives (these phenomena can also cause problems such as delamination and incompatibility). On the other hand, plasma treatment has many advantages, such as low energy consumption and high efficiency with minimal environmental impact. Plasma treatment benefits from speed and control of the process [19,20].

Correspondingly, the modification of polyolefins’ properties can be performed by different methods that concurrently alter the adhesive and mechanical behavior, as well as temperature stability and chemical resistance. Out of these various methods, especially radiation cross-linking, is nowadays gaining gradually increasing interest from industry [21,22,23,24]. Kopal et al. [25] proved that the 150 kGy dose of radiation improved the mechanical properties of PS PMX3 (blend of melamine resin, phenol formaldehyde resin, and nitrile rubber), especially the tensile strength, stress at break and elasticity modulus. In addition to the research papers dealing with the positive effect of radiation cross-linking on mechanical properties, temperature stability, and chemical resistance [26,27,28], studies were conducted in the area of radiation cross-linking effect on the polymers’ surface layer properties. This problem was investigated extensively in our previous publications [8,29,30,31], which covered the research of non-polar polymers’ surface layer properties modified by electron radiation. It was proved that this kind of modification has a positive influence on the overall load capacity of the adhesive bond between the materials with lower adhesiveness.

Currently, the most widespread method of surface layer modification, which focuses on improving the adhesive properties in industrial applications, is still plasma treatment, which has very low energy consumption against β^−^ radiation treatment The energy consumption of β^−^ radiation must be large enough to cause cross-linking of the material, which is usually the primary effect. However, using radiation cross-linking to modify the surface layer properties of polymer materials carries a significant benefit, since besides the improved surface layer behavior, enhanced mechanical properties, as well as temperature stability and chemical resistance, can be gained. The radiation penetrates the entire material mass, while the low output plasma affects only the surface. Furthermore, the plasma has to be in direct contact with the surface, while the specimens exposed to the beta radiation can be stored in a container during the procedure. This fact can be quite beneficial for industrial applications. A paper comparing the effectiveness of plasma treatment and the technology of radiation cross-linking on the modification of polymer materials’ surface layer properties, e.g., surface energy, wetting and adhesive capabilities, has not yet been published.

Thus, this research focuses on the comparison of the different technologies, i.e., cold atmospheric-pressure plasma and radiation cross-linking, which are both usable for the modification of the polymer materials’ surface layers. The cross-linking within the polymer materials was in this case instigated by the β^−^ radiation. The main goal of this work is to compare the effect of the aforementioned technologies on the wetting contact angle, free surface energy, and its polar component and the adhesive properties of the polymer material selected from the group of polyolefins.

## 2. Materials and Methods

### 2.1. Material and Sample Preparation

The comparison of aforementioned surface modification methods was done on specimens prepared from polypropylene that is commercially known as PP V-PTS-CREALEN-EP2300L1*M800, which was supplied by PTS (Adelshofen, Germany). This polymer belongs to the group of polyolefins. Materials belonging to the polyolefin group can be characterized by their non-polarity and subpar adhesive properties, which leads to non-optimal adhesion, unless it is preceded by surface alteration. To ensure cross-linking, PP contained 4% triallylisocyanurate as its cross-linking agent. The entire granulate preparation process with the cross-linking agent was performed by the PTS Plastic Technology Service company.

The test samples were produced by an Arburg Allrounder 470e (Loßburg, Germany) injection molding machine. Process parameters, which can be seen in Table 1, were set according to the recommendation of the material’s manufacturer. The shape and dimensions of the specimens that were later joined by adhesive bonding was governed by the CSN EN 1456 standard [32].

### 2.2. Cold Atmospheric-Pressure Plasma Surface Treatment

**Figure 1:**
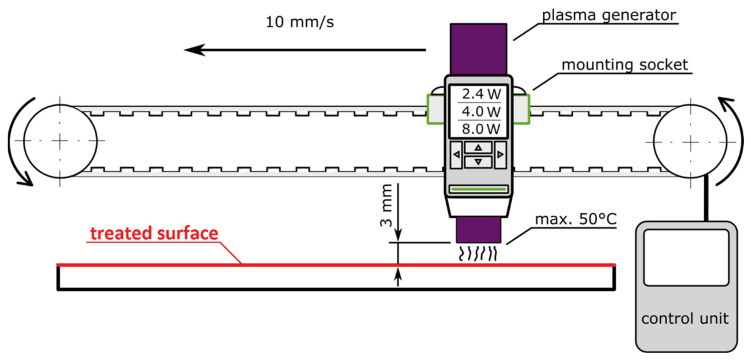
Plasma treatment experimental setup.

The cold atmospheric-pressure plasma treatment of the surface was done by the piezobrush^®^ PZ3 Professional Set (Regensburg, Germany). The main part of this plasma device is the piezo-electric plasma generator (CarePlas™), which is basically a high voltage discharge device used to generate cold atmospheric plasma with temperatures lower than 50 °C. The advantage of this solution is the low entry voltage, which can be transformed with maximum efficacy into a powerful electric field [33]. Afterwards, the field dissociates and ionizes the ambient processing gas, which is ambient air. The ambient temperature and relative humidity were set to following values during the cold plasma treatment: (23 ± 2) °C and (55 ± 5)%. The test samples were treated by three magnitudes of plasma: 2.4 W, 4 W, and 8 W. The plasma generator has been mounted to the socket and via timing belt gear moved over the treated surface in a linear direction with 3 mm clearness between the plasma generator and treated surface. The speed of the whole system has been controlled with a stepper motor and set to 10 mm/s (see Figure 1).

### 2.3. β^−^ Radiation Surface Treatment

The second tested method for the modification of the studied surface layers was β^−^ (accelerated electrons) radiation. This was done under standard atmospheric conditions and room temperature in cooperation with BGS Beta-Gamma Service, located in Germany. The source of accelerated electrons was a Rhodotron high-voltage accelerator, which presented the maximum energy of 10 MeV (Tongeren, Belgium). The range of the dosages was set, in compliance with experience gained from industrial practice, to 33, 66, and 99 kGy. Each accelerator cycle exposed the test sample to the radiation dose of 33 kGy. The adequate radiation dose was determined by a Nylon FTN 60-00 dosimeter (Goleta, CA, USA). The analysis of absorbed radiation dose by the dosimeter was performed with a Genesys 5 spectrophotometer, in accordance with the ASTM 51261 standard [34].

### 2.4. Adhesives and Construction of Bonded Joints

Three groups of commercially available adhesives, cyanoacrylate-, acrylate-, and epoxide-based, were used to create adhesive bonds (see Table 2).

**Figure 2:**
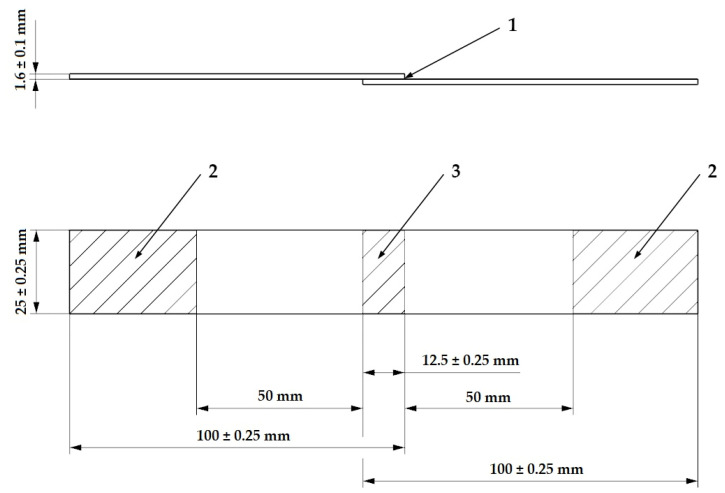
Bonded joint: (1) adhesive layer, (2) area of test machine grips, and (3) shear area [32].

The adhesive bonds were created by intermediate layer between the treated and virgin samples. The creation of the adhesive bond was given by CSN EN 1456 standard [32], and its shape and dimensions can be seen in Figure 2. Constant thickness was assured by supports, which were placed in between the specimens.

### 2.5. Wetting Contact Angle Measurements

**Figure 3:**
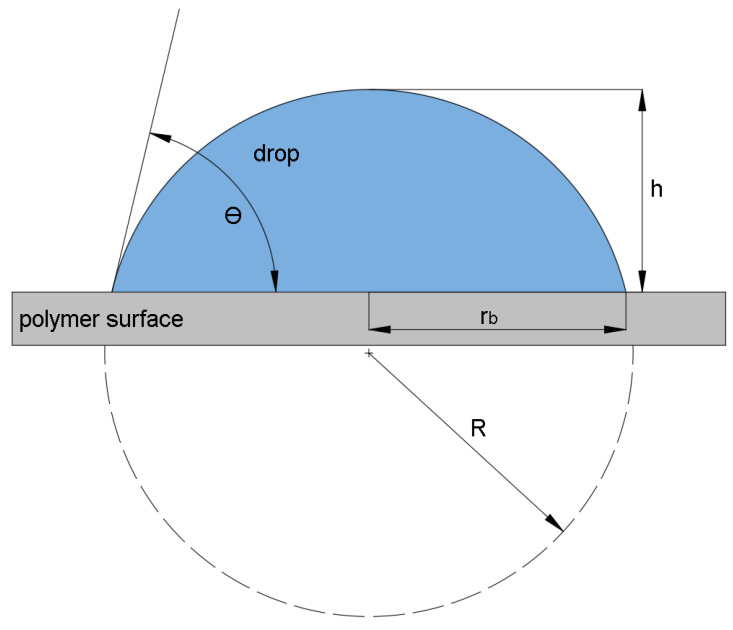
Droplet profile analysis: θ—wetting contact angle, h—droplet height, r_b_—droplet radius at contact point, and R—entire droplet radius.

The wetting contact angle, which characterizes the wetting capability of surfaces, was measured by sexagonal system in cooperation with a See System device (Advex Instruments, Brno, Czech Republic). The measurements were done according to the CSN EN 15802 standard [35]. Three reference liquids were used (distilled water, glycerin and ethylene glycol). Each reference liquid had a varying value of surface tension: 72.8 mJ/m^2^ for distilled water, 64 mJ/m^2^ for glycerin, and 48 mJ/m^2^ for ethylene glycol [36]. The testing was done 24 h after the surface treatment and 15 measurements were done for each liquid and every sample. The drops of the reference liquids were applied on the polymers’ surface layer by micropipette with volume of 4 µL per one applied drop. The drop profile was analyzed, which subsequently led to the determination of the wetting contact angles, as can be seen in Figure 3.

The height of the drop (h) and the radius at the point of impingement (r_b_) were used to calculate the wetting contact angle (θ) through the following set of equations [8,37]:

(1) h=R(1−cosθ),

(2) $$r_{b}=\mathrm{Rsin}\theta ,$$

and:

(3) $$\frac{h}{r_{b}}=\frac{1-\cos\theta }{\sin\theta }=\tan\left(\frac{\theta }{2}\right).$$

### 2.6. Determination of Surface Energies

To determine the free surface energy of the tested polypropylene, the Owens–Wendt–Rabel–Kaelble (OWRK) regressive method was used [38,39,40]. This method, which uses the measured wetting contact angles in order to determine the free surface energy, is commonly utilized for these applications and it was derived from the Fowkes method [41]. The OWRK method states that the surface energy is given by the sum of dispersive (γ_d_) and polar component (γ_p_). In order to determine the surface energy of liquids (γ_1_) and solids (γ_s_), following equations were used [38,39,40]:

(4) $$\gamma_{l}=\gamma_{l}^{d}+\gamma_{l}^{p},$$

(5) $$\gamma_{s}=\gamma_{s}^{d}+\gamma_{s}^{p},$$

(6) $$\gamma_{l}\left(1+\cos\theta \right)=2\sqrt{\gamma_{s}^{d}\gamma_{l}^{d}}+2\sqrt{\gamma_{s}^{p}\gamma_{l}^{p}},$$

and:

(7) $$\left(\gamma_{l}^{d}+\gamma_{l}^{p}\right)\left(1+\cos\theta \right)=2\sqrt{\gamma_{s}^{d}\gamma_{l}^{d}}+2\sqrt{\gamma_{s}^{p}\gamma_{l}^{p}}.$$

Aforementioned equations were subsequently edited into linear equations. The final values of free surface energy were then determined by linear regression (see Equation (8)), which required the use of three reference liquids for its calculations. This solution can contribute to significant reduction of errors caused by inappropriate combination of reference liquids [38,39,40,42].

(8) $$\frac{\left(1+\cos\theta \right)\gamma_{l}}{2\sqrt{\gamma_{l}^{d}}}=\sqrt{\gamma_{s}^{p}}\sqrt{\frac{\gamma_{l}^{p}}{\gamma_{l}^{d}}}+\sqrt{\gamma_{s}^{d}}$$

### 2.7. Measurement of Load-Bearing Capacity of Bonded Joints

To establish the effect of both surface treatment methods on the final load bearing capacity of adhesive bonds, the shear strength of the bond was measured by tensile testing. This was performed on a Zwick 1456 (ZwickRoell, Ulm, Germany) universal testing machine with a crossbar speed of 50 mm per minute. The specimen, in form of adhesive bond (see Figure 2), was symmetrically placed into the grips and the distance between them was set to (50 ± 1) mm. In order to ensure the force was applied in the plane of adhesive bond, levelling pads were used within the area of the grips. The measurements were done in room temperature (23 °C) and the gained data was subsequently evaluated by Test Expert software (version 2, ZwickRoell, Ulm, Germany).

### 2.8. Fourier-Transform Infrared (FTIR) Spectroscopy

FTIR spectra of PP samples were recorded using a Nicolet iS50 FTIR (Thermo Scientific™) equipped with attenuated total reflection (ATR) technique and a pure ATR diamond crystal. The spectra were collected at resolution of 4 cm^−1^, using 60 spectrum accumulation. Omnic software 9.2 was then utilized for data processing, baselines were corrected manually and the average of five spectra was used for results evaluation.

### 2.9. Scanning Electron Microscopy (SEM)

To explore and compare impact of different treatment methods on sample surface the electron microscopy method was used. A Zeiss EVO MA15 electron microscope (Carl Zeiss AG, Oberkochen, Germany) and a Quorum Q150R ES sputter device (Quorum Technologies, Lewes, UK) equipped with a sputter thickness monitor were used for the mentioned purpose. The analyzed polymer samples were plated by sputtering a 5 nm thick gold layer. The measurement was performed in high vacuum mode (<10^−3^ Pa) and the image was then created by processing data from secondary electron detector. The working distance was 8.5 mm, the probe current was 200 pA, and the accelerating voltage 20 kV.

## 3. Results

This study is focused on the comparison of two different methods for surface layer modification in terms of their influence on surface layer properties and the final load bearing capacity of adhesive bonds created between polymers selected from the polyolefin group. Polypropylene was chosen as the tested material, specifically due to its low adhesiveness, which is a typical feature of the polyolefin group. The first method used cold atmospheric-pressure plasma with outputs of 2.4, 4, and 8 W to modify the surface, while the second method used β^−^ radiation with doses of 33, 66, and 99 kGy to do the same. Every measured result was put into following tables and graphs and subsequently presented in the form of arithmetic averages with appropriate standard errors of the mean. Every measurement was repeated 15 times.

### 3.1. Surface-Layer Properties

**Figure 4:**
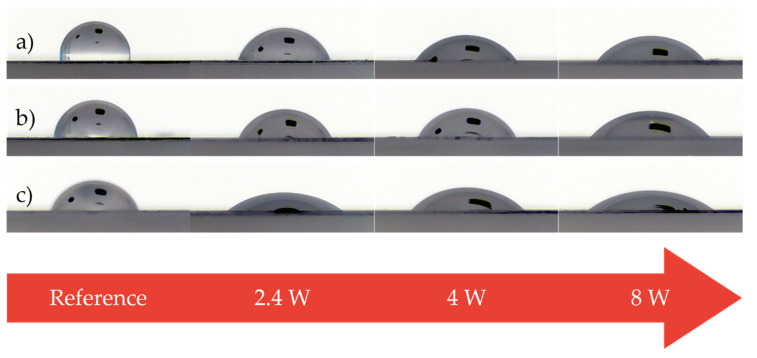
Liquid droplets on surfaces of test material (depending on plasma power): (**a**) distilled water, (**b**) glycerin, and (**c**) ethylene glycol.

**Figure 5:**
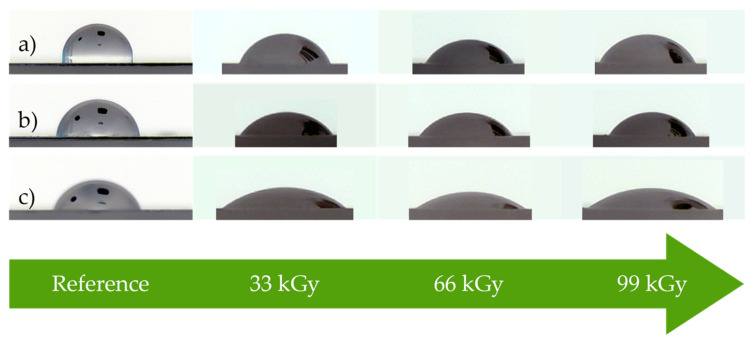
Liquid droplets on surfaces of test material (depending on radiation dose): (**a**) distilled water, (**b**) glycerin, and (**c**) ethylene glycol.

Effects of both methods of modification of the surface layer properties were evaluated by the wetting contact angles, free surface energy and its polar component. In the previous studies [9,11], it was found that the value of wetting contact angle is quite an important factor for the process of adhesive bonding, as the main assumption for smooth adhesion with high strength is the sufficient wetting of the adherend’s surface by adhesive. The changes in the wetting contact angles of polypropylene after the plasma treatment can be seen in Table 3 and Figure 4, while the data obtained following the β^−^ radiation treatment are shown in Table 4 and Figure 5. Properties of the non-altered surface were taken as the reference value.

The measurements displayed in Table 3 and Figure 4 show, that the surface of the PP treated by the cold plasma had a significantly better wetting, which was caused by the decrease of the wetting contact angles. This decrease was noted with each applied reference liquid. Non-altered surface was measured to have the highest values of the wetting contact angles; 88.7° for distilled water, 81.6° for glycerin, and 67.1° for ethylene glycol. A considerable decline in wetting contact angles was already found in surface treated by plasma with output 2.4 W; however, the lowest values were measured in specimens, for whom the output was 8 W; 46.6° for distilled water, 46.8° for glycerin and 34.7° for ethylene glycol. As can be seen, the values were decreased by at least 40% for every reference liquid.

Table 4 and Figure 5 show, that using β^−^ radiation treatment on the PP surface has a similar positive effect on the observed properties, i.e., improved wetting, which leads to lower wetting contact angles. This decline was observed for every applied reference liquid. The highest values of wetting contact angle for the virgin surface were measured as follows: 88.7° for distilled water, 81.6° for glycerin and 67.1° for ethylene glycol. A significant decrease of wetting contact angle was found even in specimen irradiated by 33 kGy; however, the lowest values of this observed property was seen in samples exposed to radiation dosage of 66 kGy. The wetting contact angle of these test subjects was 59.8° for distilled water, 56.9° for glycerin and 37.1° for ethylene glycol, which sums up to at least a 30% reduction for each reference liquid.

Surface exposure to 99 kGy of β^−^ radiation proved to have no positive effect, on the contrary a slight decrease in wetting contact angle was observed for all reference liquids, as can be seen in Table 4 and Figure 5. This phenomenon might be related to the cross-linking parameter G(X) and chain fission parameter G(S). Parameter G is a value that is used to determine the chemical gain, which is created due to the radiation. This parameter is defined by chemical gain from reacting molecules in 100 eV of absorbed energy. Cross-linking and chain fission are two opposite processes, which always coexist due to irradiation. The overall effect of irradiation depends on which of these processes prevails at the given time. If parameter G(X) > G(S), then the result is cross-linking. However, if the parameter G(X) < G(S), then the result is degradation. Both of these parameters increase with higher radiation dosage. However, the parameter G(S) generally increases quicker than G(X) in polymer materials [21,22]. In case of this study, the parameter G(X) was higher than G(S) up until the radiation dosage of 66 kGy, which resulted in cross-linking and thus improved wetting. Since the parameter G(S) increased quicker than its counterpart, the balance turned to G(S) > G(X) for the specimen irradiated by 99 kGy, which resulted in degradation prevailing over cross-linking. Due to this process, the wetting of the surface declined. This phenomenon was described and carefully observed in previous research [8] that was published in 2019. In this article, the dependence of gel content on absorbed dose of radiation in PP was observed. It was found, that the gel content in PP increased up to the radiation dosage of 66 kGy. Doses higher than that resulted in minor decrease in gel content, which corresponds with the wetting results.

The decrease of wetting contact angles, which was observed in specimens treated by both surface modifying methods, tightly corresponds with changes of the free surface energy. The theory of wetting [11] states that in order to create a quality adhesive bond, the adherend should possess higher surface energy than the applied adhesive, which is also one of the reasons why the surface of the tested PP had to be modified before the adhesive bonding. Changes of the free surface energy (γ_s_) and its polar (γ_p_) and dispersive component (γ_d_), which were instigated by the plasma treatment, can be seen in Table 5, while the changes to the same parameters triggered by the β^−^ radiation can be found in Table 6. The surface energy of the virgin material was taken as a reference point.

The measurements that were made on surfaces modified with cold plasma indicate that the free surface energy rose with increasing plasma output, as shown in Table 5. This had significantly improved the surface layer adhesive properties of the specimen. The highest growth was observed in test samples treated with plasma with maximum output, i.e., 8 W. In this case, the free surface energy increased from 22.6 mJ/m^2^ to 54.5 mJ/m^2^, which could be translated to approximately 140% growth in comparison with reference value.

Similar results, i.e., increased values of free surface energy and improved adhesive properties, could be observed in surfaces that were modified by β^−^ radiation, as can be seen in Table 6. The best improvement was found in specimen irradiated by 66 kGy, in which the values of free surface energy rose from 22.6 mJ/m^2^ to 41.1 mJ/m^2^. When compared with reference value, there was an approximate increase of 80%.

The trend observed with free surface energy was comparable to its polar component, which can be found in Table 5 and Table 6. Following the plasma treatment of the surface, the values of surface energy polar component in PP increased from 6.7 mJ/m^2^ measured in reference sample to 47.3 mJ/m^2^ found in specimen that was treated by cold plasma with 8 W output, which was an increase of 700%. When the β^−^ radiation was used to modify the surface, the polar component of the free surface energy climbed to 27.8 mJ/m^2^, which was a 300% increase in comparison with the reference value.

### 3.2. Load-Bearing Capacity of Bonded Joints

**Figure 6:**
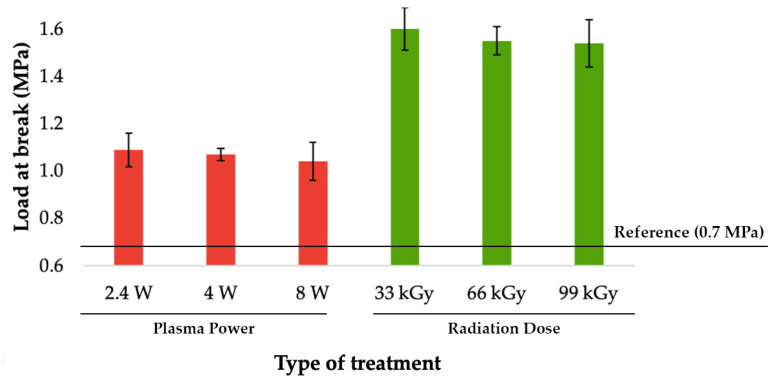
Load-bearing of adhered joints for cyanoacrylate adhesive (depending on plasma power and radiation dose).

**Figure 7:**
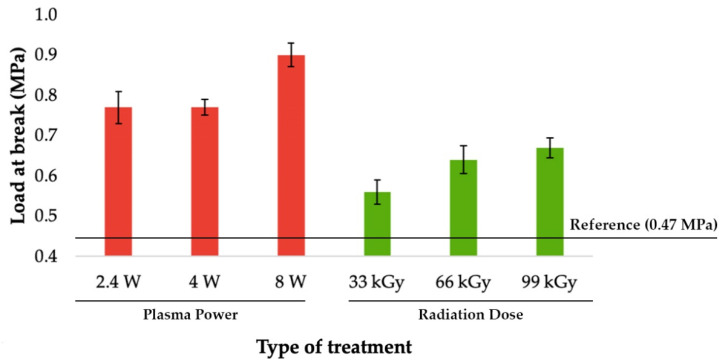
Load-bearing of adhered joints for two-component acrylate adhesive (depending on plasma power and radiation dose).

**Figure 8:**
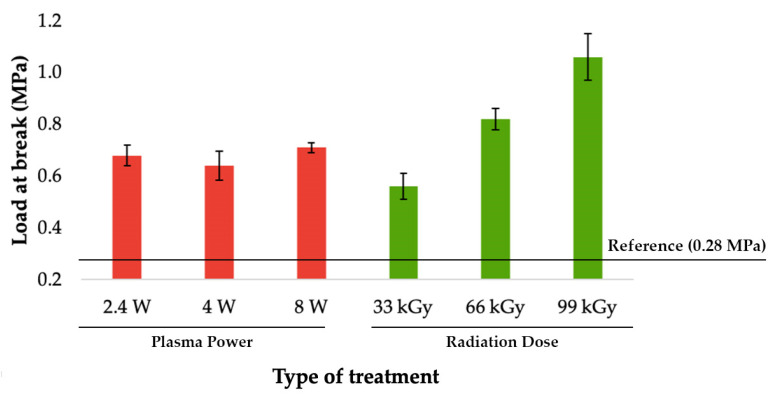
Load-bearing of adhered joints for two-component epoxide adhesive (depending on plasma power and radiation dose).

The most important value of adhesive bonding between two polyolefins, is the load bearing capacity of bonded joints. This parameter was determined by measuring the shear strength of the bond with tensile test. As can be seen in Figure 6, Figure 7 and Figure 8, the specimens’ surface treated by both aforementioned methods, i.e., cold plasma and β^−^ radiation, displayed a significant increase of the bond’s strength for every type of tested adhesive, i.e., cyanoacrylate-, acrylate-, and epoxide-based. The strength of the adhesive bond connecting the virgin materials was taken as a reference point.

Among the specimens that were treated with cold plasma and connected with cyanoacrylate adhesive; the biggest growth of bond’s strength was found in test subject exposed to the plasma with lowest output, as can be seen in Figure 6. This sample was treated by plasma with output 2.4 W and its bond strength rose up to 1.09 MPa, which is an increase of approximately 56% in comparison with the reference point. On the other hand, higher plasma outputs (4 W and 8 W) did not produce better results, and its application led to a slight decrease of the bond’s strength.

Likewise, the specimens that were modified by β^−^ radiation and connected with cyanoacrylate had their biggest upsurge of bond’s strength found in samples irradiated by lowest dosage (33 kGy), due to which the strength rose to 1.6 MPa, which is approximately a growth of 130% in comparison with the reference point (see Figure 6). Higher dosages of radiation displayed a similar trend to the cold plasma treatment and, as such, had a slightly negative effect on the bond’s strength. However, with increasing radiation dose or plasma power, the load-bearing capacity of bonded joints decreases slightly, but these values are within the standard deviation.

The second type of bonding agent, two-component acrylate adhesive, displayed the best results when it was applied to specimen modified by plasma with the highest output (8 W). The same trend, as shown in Figure 7, were found in samples exposed to the β^−^ radiation. For the former case, the strength of the bond rose to 0.9 MPa, which is approximately a growth of 90% in comparison with the reference point. On the other hand, the latter case provided a slightly lower increase, as the bond’s strength was 0.65 MPa, which is an approximate increase of 40% in comparison with the reference point, as can be seen in Figure 7.

The last type of applied bonding agent was the two-component epoxy adhesive. The highest strength of the bond created by aforementioned adhesive was found in specimens that were treated by cold plasma with output of 8 W. However, given the size of the standard deviation, it can be stated that the strength of all joints was almost the same and shows an increase of 150% compared to the reference value (see Figure 8). Correspondingly, the strongest bond created by the two-component epoxy adhesive was measured in samples exposed to maximal radiation (99 kGy). As displayed in Figure 8, this combination improved the strength of the bond to 1.1 MPa, which is a significant growth of 290% when compared with the reference point.

## 4. Discussion

This study dealt with comparison of influence that radiation cross-linking and plasma treatment have on surface properties and final load bearing of adhesive bonds on PP. The chosen material is characteristic for its non-polar behavior and subpar wetting of the surface, due to which it tends to create adhesive bonds with very poor quality. This problem can be solved with suitable surface modification method. In this research, the type of radiation cross-linking was β^−^ radiation, while the type of plasma was cold atmospheric-pressure plasma.

The results of the measurements uncovered, that both of the tested modification methods had a very positive influence on the wetting ability of tested surfaces, which was shown by the decline of wetting contact angles. The wetting contact angles, after the application of the cold plasma treatment with 8 W output, were reduced by at least 40% for every reference liquid (see Table 3 and Figure 4). On the other hand, the wetting contact angles of the surface treated with 66 kGy of β^−^ radiation decreased by at least 30% for each reference liquid (see Table 4 and Figure 5). A significant improvement of wetting capabilities corresponds with the increase of free surface energy and its polar component (see Table 5 and Table 6). The aforementioned properties were calculated from the measurements of wetting contact angles. The creation of reliable adhesive bonds is conditional on the adherend having higher surface energy than the adhesive [9,11]. Polypropylene, which was tested in this research, belongs to a group of polymer materials with very low surface energy. The results indicate that high surface energy can be reached by both cold plasma and β^−^ radiation modification. According to the previous publications [9,11], the high energy surfaces of polymer materials must have the surface energy higher than 40 mJ/m^2^. In case of this study, the condition was successfully satisfied for both of the methods of modification (see Table 5 and Table 6).

**Figure 9:**
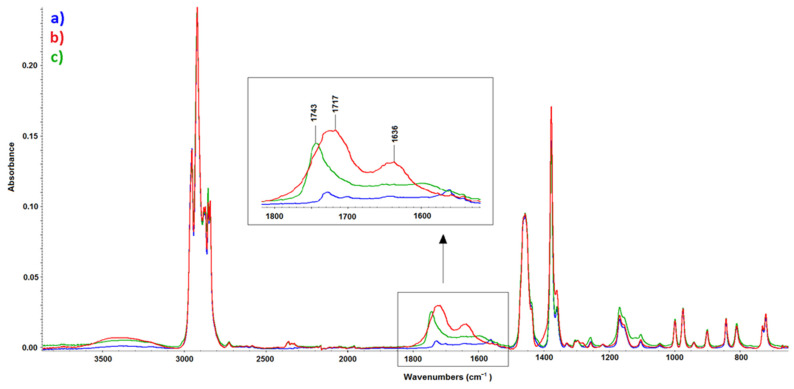
Infrared spectra of (**a**) PP, untreated, (**b**) PP, after plasma treatment (8 W), and (**c**) PP, after β^−^ radiation treatment (99 kGy).

The main factor that influences the surface property changes, was most likely the oxidation of PP caused by both cold plasma and β^−^ radiation. The reaction/interaction led to the increase of functional carbonyl group in the PP polymer chain [12,13,43,44,45,46]. Thus, this assumption was investigated by infrared spectroscopy. Compared the spectra of unmodified PP (Figure 9a) to spectra of modified PP by plasma (Figure 9b) or β^−^ radiation (Figure 9c) some differences were found. In cases of PP with surfaces modified by the highest radiation dosage and plasma output, characteristic absorption bands in the range of 1850 cm^−1^–1600 cm^−1^ were found. This observation indicated/confirmed expected formation of carbonyl functional groups in PP polymer chains [47]. In addition, changes in spectra in the range from 3600 to 3100 cm^−1^ suggest the formation of hydroxyl functional groups in case of treated samples, i.e., (b) and (c) [23]. These findings correspond with previously realized studies, which were focused on the changes of chemical composition of surfaces following the exposure to β^−^ radiation [8] and plasma treatment [12,13,15,16,17,18].

**Figure 10:**
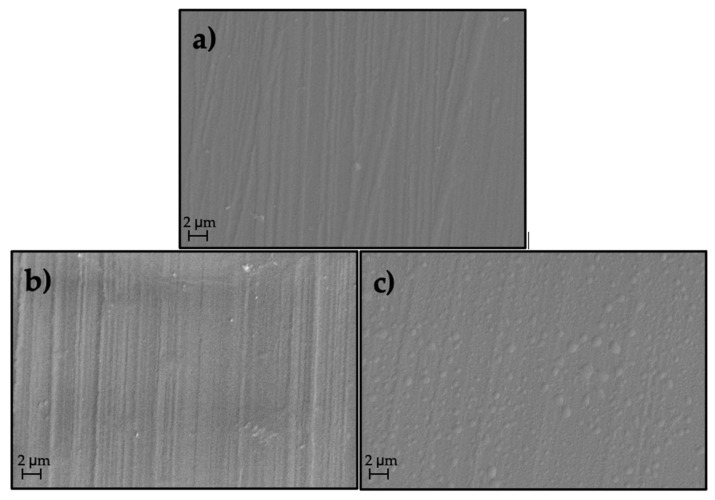
SEM micrographs of (**a**) PP, untreated, (**b**) PP, after β^−^ radiation treatment, and (**c**) PP, after plasma treatment.

In addition to the changes to the chemical composition, material undergoing plasma treatment is also etched, as a consequence of plasma flow impingement on the surface. This behavior increases the surface roughness and supports the polar properties [13]. In order to determine the changes of the morphology that were induced by both types of applied modification, the scanning electron microscopy (see Figure 10) was used. The surface of the specimens exposed to the highest β^−^ radiation and plasma output were observed.

Figure 10b,c show the change in the surface structure (surface morphology) of PP samples treated with β^−^ radiation and plasma compared to the original material (see Figure 10a).

**Figure 11:**
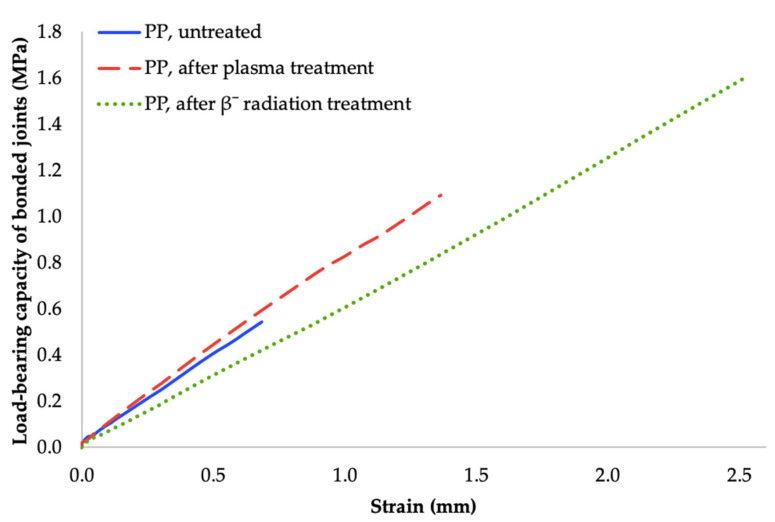
Dependence of bonded-joint load-bearing capacity of adhered joints on strain (cyanoacrylate adhesive).

As a consequence of the aforementioned changes observed in surface layers, the load bearing of adhesive bonds significantly increased. Regarding the use of cyanoacrylate adhesive; the most suitable method appeared to be the β^−^ radiation with dosage of 33 kGy that increased the bond’s strength to 1.6 MPa, which is an approximate improvement of 130% in comparison with non-altered surface. Likewise, the strength of the bond between materials exposed to plasma treatment increased; nonetheless the improvement was lower than the previous one by approximately 50% (see Figure 6 and Figure 11).

**Figure 12:**
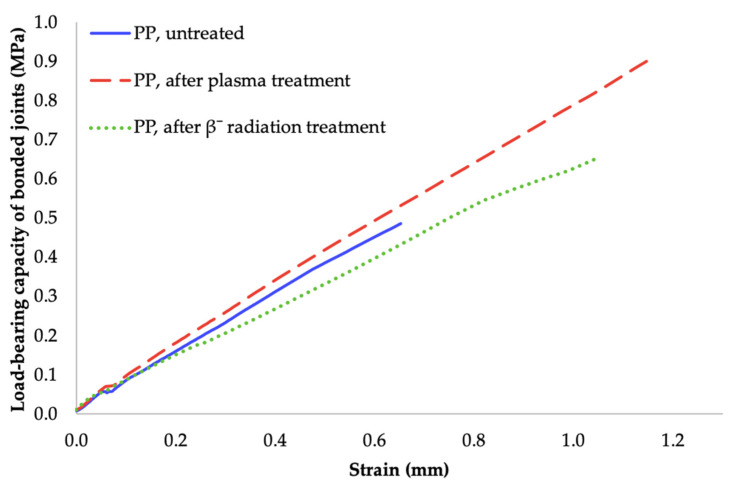
Dependence of bonded-joint load-bearing capacity of adhered joints on strain (two-component acrylate adhesive).

The most beneficial method for the use of two-component acrylate adhesive appeared to be the cold plasma treatment with output of 8 W. This modification improved the bond’s strength to 0.9 MPa, which is roughly an increase of 90% in comparison with the surface of virgin material. When compared with the results of β^−^ radiation treatment, the strength of the bond was about 40% higher for the materials exposed to cold plasma (see Figure 7 and Figure 12).

**Figure 13:**
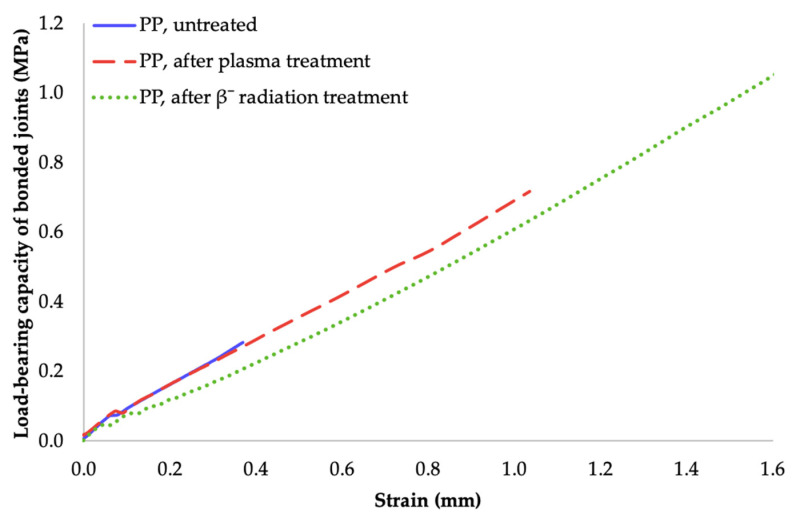
Dependence of bonded-joint load-bearing capacity of adhered joints on strain (two-component epoxide adhesive).

In the case of two-component epoxy adhesive application, the most suitable method proved to be β^−^ radiation with dosage of 99 kGy. Due to this treatment, the bond’s strength increased to 1.1 MPa, which is approximately 290% improvement in comparison with surface of the unaltered material. While the bond’s strength was also significantly improved by the cold plasma treatment, the gains from this method were inferior to those of β^−^ radiation, approximately by 60% (see Figure 8 and Figure 13).

## 5. Conclusions

The tests conducted in this research led to the following conclusions:

- β^−^ radiation and cold plasma treatment had a positive effect on both the wetting and the surface energy of the tested material;
- the adhesive properties of the polymer specimens were significantly improved by both β^−^ radiation and cold plasma treatment;
- the results proved, that β^−^ radiation is at least on the similar level of effectiveness as plasma treatment, as far as improvements of adhesive bond’s strength and adhesiveness of PP are concerned; and
- two out of the three tested bonding agents, i.e., cyanoacrylate and two-component acrylate adhesive, provided higher bond strength when applied to the surface modified by β^−^ radiation.

From the application point of view, the future research in this area should be focused on the time stability of the surface layer properties, which were gained by the tested modification methods.

## Figures and Tables

**Table 1:** Injection molding parameters.

**Processing Conditions**	**PP**
Injection Rate (mm/s)	50
Injection Pressure (MPa)	80
Injection Time (s)	0.5
Holding Pressure (MPa)	40
Holding Time (s)	10
Cooling Time (s)	40
Mould Temperature (°C)	50
**Plastic Unit Temperature Bands**	
Zone 1 (°C)	210
Zone 2 (°C)	220
Zone 3 (°C)	230
Zone 4 (°C)	240

**Table 2:** List and designation of adhesives used.

Adhesive Group	Adhesive Manufacturer	Adhesive Designation
Cyanoacrylate Adhesive	3M	PR100
Two-Component Acrylate Adhesive	3M	DP8805 NS
Two-Component Epoxide Adhesive	3M	DP100

**Table 3:** Wetting contact angles of PP materials (depending on plasma power).

Liquid	Reference	Plasma Power (W)		
		2.4	4	8
Distilled Water	(88.7 ± 0.3)°	(71.9 ± 0.1)°	(55.8 ± 0.3)°	(46.6 ± 0.2)°
Glycerin	(81.6 ± 0.2)°	(63.2 ± 0.2)°	(55.4 ± 0.4)°	(46.8 ± 0.2)°
Ethylene Glycol	(67.1 ± 0.2)°	(37.1 ± 0.1)°	(41.1 ± 0.4)°	(34.7 ± 0.3)°

**Table 4:** Wetting contact angles of PP materials (depending on radiation dose).

Liquid	Reference	Radiation Dose (kGy)		
		33	66	99
Distilled Water	(88.7 ± 0.3)°	(69.3 ± 0.3)°	(59.8 ± 0.1)°	(61.9 ± 0.4)°
Glycerin	(81.6 ± 0.2)°	(58.3 ± 0.5)°	(56.9 ± 0.4)°	(57.1 ± 0.6)°
Ethylene Glycol	(67.1 ± 0.2)°	(42.2 ± 0.4)°	(37.1 ± 0.2)°	(37.3 ± 0.3)°

**Table 5:** Free surface energy and its elements for PP (depending on plasma power).

Free Surface Energy and Its Elements (mJ/m^2^)	Reference	Plasma Power (W)		
		2.4	4	8
γ_s_	22.6	37.1	45.1	54.4
γ_s_^p^	6.7	10.9	36.6	47.3
γ_s_^d^	15.9	26.2	8.5	7.1

**Table 6:** Free surface energy and its elements for PP (depending on radiation dose).

Free Surface Energy and Its Elements (mJ/m^2^)	Reference	Radiation Dose (kGy)		
		33	66	99
γ_s_	22.6	36.7	41.1	39.6
γ_s_^p^	6.7	14.9	27.8	24.2
γ_s_^d^	15.9	21.8	13.3	15.4

## Data Availability

MDPI is committed to supporting open scientific exchange and enabling our authors to achieve best practices in sharing and archiving research data. We encourage all authors of articles published in MDPI journals to share their research data. More details in section “MDPI Research Data Policies” at https://www.mdpi.com/ethics.
